# MiR‐145 inhibits human colorectal cancer cell migration and invasion via PAK4‐dependent pathway

**DOI:** 10.1002/cam4.1029

**Published:** 2017-04-24

**Authors:** Nengquan Sheng, Gewen Tan, Weiqiang You, Hongqi Chen, Jianfeng Gong, Di Chen, Huizhen Zhang, Zhigang Wang

**Affiliations:** ^1^ Department of General SurgeryShanghai Jiao Tong University Affiliated Sixth People's HospitalShanghai200233China; ^2^ National Key Laboratory of Science and Technology on Nano/Micro Fabrication TechnologyResearch Institute Micro/Nano Science and TechnologyShanghai Jiao Tong UniversityShanghai200240China; ^3^ Department of PathologyShanghai Jiao Tong University Affiliated Sixth People's HospitalShanghai200233China; ^4^ Department of General SurgeryShanghai Jiao Tong University Affiliated Sixth People's HospitalShanghai200233China

**Keywords:** colorectal cancer, cytoskeleton, migration, miR‐145

## Abstract

MicroRNA‐145 (miR‐145), as a tumor‐suppressive miRNA, has been demonstrated down‐regulated in colorectal cancer (CRC) cells, and could inhibit CRC cells growth. However, the molecular pathway in which miR‐145 modulates CRC malignant transformation has not been fully revealed. Here, we reported an intense correlation between the expressions of PAK4 and miR‐145 in human CRC cell lines. Transwell assay verified overexpression of miR‐145, as well as knockdown of PAK4, significantly suppressed cell migration and invasion ability. The impaired migration and invasion ability of SW1116 cells was affected through the down‐regulation of phosphorylation level of LIMK1 and cofilin in a PAK4‐dependent manner. Collectively, we have demonstrated that miR‐145 suppressed CRC migration and invasion through PAK4 pathway, which provides an attractive microRNA‐based therapeutic target for CRC.

## Introduction

Colorectal cancer (CRC) is characterized by rapid metastasis 1 and ranks the fourth leading cause of cancer mortality worldwide 2. Although some improvements have been made in conventional therapies for CRC, including surgical operation, over one‐third of patients with CRC develop metastatic diseases mainly due to metastasis 3, 4. Therefore, understanding of the molecular mechanisms associated with CRC metastasis is urgently required to help provide more effective treatments against CRC. MicroRNAs (miRNAs, miRs), a class of endogenous short non‐coding RNAs consisting of 17‐25 nucleotides 5, has been reported to regulate tumorigenesis and metastasis and act as oncogenes or tumor‐suppressive genes depending on the targets they regulate^6^.

MiR‐145 was identified as a tumor‐suppressive miRNA and firstly found to be down‐regulated in CRC 7. Further studies have demonstrated miR‐145 participates in CRC by regulating a series of certain gene expressions participated in tumorigenesis and metastasis 8, 9. P21‐activated kinases 4 (PAK4) is identified as a member of PAK family and classified as group II PAKs lately 10, which is essential in cell cytoskeletal reorganization both prerequisite steps for cell migration. Overexpression of PAK4 is found in many cancers and considered as an important oncogene that promotes migration [11, 12, 13, 14, 15, 16. Our previous study has indicated PAK4 is a novel target of miR‐145 and its suppression involved in MAPK pathway leading to CRC cell growth inhibition{Wang, 2012 #17]. However, the molecular mechanism by which miR‐145 modulates CRC metastasis is relatively underexplored.

To investigate the critical function and mechanism of miR‐145‐PAK4 signaling pathway in CRC metastasis, we firstly determined the expression levels of miR‐145 and PAK4 in several CRC cell lines. Then we studied the effects of miR‐145 and PAK4 on CRC cell migration and invasion ability, by overexpression of miR‐145, as well as knockdown of PAK4. Furthermore, we revealed miR‐145 suppressed CRC migration and invasion through PAK4 pathway, providing a potential therapeutic approach for CRC.

## Materials and Methods

### Cell lines and culture

Seven Human CRC cell lines, DLD‐1, HCT116, SW1116, SW620, SW480, Lovo, RKO, and HT29, were obtained from Cell Bank of Chinese Academy of Sciences (Shanghai, China). SW1116 cells was grown in Dulbecco's modified Eagle's medium (Hyclone, SH30243. 01B). DLD‐1, HCT116, SW480, Lovo and RKO cells were cultured in RPMI‐1640 medium (Hyclone, SH30809. 01B). SW620 cells was grown in L‐15 medium (Sigma L1518) and HT29 cells was grown in MCCOYS’ 5A (Sigma M9309). All media were supplemented with 10% heat‐inactivated fetal bovine serum (FBS; BI 04‐001‐1A) in a humidified 5% CO_2_ atmosphere.

### Plasmid construction, packaging, and infection

To study the relation between miR‐145, PAK4 expression and CRC, the sequence of miR‐145 (MI0000461) was cloned into the lentiviral vector pCDH vector (Shanghai Hollybio, China) between EcoRI and BamHI restriction enzymes to generate miR‐145 overexpressed plasmid, which was termed as oemiR‐145. For PAK4 knockdown plasmid construction, short hairpin RNA (shRNA: 5’‐ GATCCGCCACAGCGAGTATCCCATGACTCGAGTCATGGGATACTCGCTGTGGCTTTTTG‐3’) targeting PAK4 (NM_001014831.2) was designed to specifically knock down PAK4 expression. Control shRNA sequence was 5’‐ GATCCTTCTCCGAACGTGTCACGTCTCGAGACGACGCACTGGCGGAGAATTTTTG‐3’. The oligos were annealed and inserted into pFH‐L vector (Holly Lab Shanghai) and constructed corresponding lentiviral particles using HEK293 cells using Lipofectamine 2000 (Invitrogen, Carlsbad, CA). The generated plasmids were referred to as shPAK4 and shCon.

Infectious lentiviruses were collected at 96 h after transfection and lentiviral particles were purified by ultracentrifugation (4000*g*) at 4°C for 10 min and filtered through 0.45 *μ*m filter. Target cells, SW1116 cells, were infected with oemiR‐145, shPAK4 and shCon, respectively, and cultured in six well plates with an inoculation density of 2 × 10^4^ cells. Successful infection was measured by calculating the cells with positive green fluorescent protein (GFP) under fluorescence microscope. The knockdown and overexpression efficiency in SW1116 cells were thereafter confirmed by quantitative real‐time polymerase chain reaction (qRT‐PCR) and western blot analysis.

### RNA extraction and quantitative PCR analysis

Total RNAs was extracted from cells using Trizol reagent (Invitrogen). Single strand cDNA was synthesized using M‐MLV reverse transcriptase kit (Promega, Madison, USA). Primers 5’‐ACACGCGTCCAGTTTTCCC‐3' (forward) and 5’‐GGTCCGAGGTATTCGCAC‐3’ (reverse) were used to detect miR‐145 expression. β‐actin's primers 5’‐GTGGACATCCGCAAAGAC‐3’ (forward) and 5’‐AAAGGGTGTAACGCAACTA‐3’ (reverse) was designed as an internal control. PCR was performed using BioRad connet Real‐time PCR platform. Total 20 *μ*L PCR reaction mixture was 10 *μ*L 2×SYBR premix ex taq, 0.8 *μ*L primers (2.5 μmol/L), 5 *μ*L cDNA and 4.2 *μ*L of ddH_2_O. The detailed PCR procedure was as follows: a denaturation step at 95°C for 1 min, 40 cycles of annealing at 60°C for 20 sec and an extension step at 72°C for 10 min. Then the relative mRNA expression of miR‐145 levels was calculated through 2^‐ΔΔCT^ formula.

### Western blot analysis

Five days after lentiviral infection, cells were harvested and lysed in 2× protein lysis buffer (10 mmol/L EDTA, 100 mmol/L Tris‐Hcl (pH 6.8), 4% SDS and 10% Glycine). The suspension was collected after centrifugation at 12,000*g* for 15 min at 4°C.Equal amounts (30 *μ*g) of proteins were run on a 10% SDS‐PAGE and subjected to electrophoresis at 50 V for 3 h on a 10 % separating‐gel. The gel was transferred to polyvinylidene difluoride (PVDF) membrane, which was then blocked with TBST containing 1% bovine serum albumin (BSA) in for 1 h and incubated with primary antibodies overnight and then incubated with secondary antibodies for 2 h. Primary antibodies used were as follows: rabbit anti‐PAK4 (1:1000, Proteintech Group, Inc., 14685‐1‐AP), rabbit anti‐LIMK1 (1:1000, Proteintech Group, Inc., 19699‐1‐AP), rabbit anti‐p‐LIMK1 (1:1000, SAB, 11126), rabbit anti‐p‐cofilin (1:1000, SAB, 11139), mouse anti‐Cofilin (1:1000, Proteintech Group, Inc., 66057‐1‐Ig) and rabbit anti‐GAPDH (1:1000, Proteintech Group Inc., 10494‐1‐AP).

### Trans‐well migration assay

Transwell assay was used to determine the motility and migration of SW1116 cells. Trypsinized SW1116 cells (1.0 × 10^5^ cells/well) were transferred into the upper chambers of the Transwell plates (8 *μ*m pore size, Millipore). The growth medium supplemented with 10% FBS was added into the bottom chamber. The cells were incubated for 48 h and then the migratory cells were stained with crystal violet after using 4% paraformaldehyde to fix them. The stained cells and dissolved crystal violet were measured using light microscope and spectrometric absorbance at 570 nm respectively.

### Matrigel invasion assay

The cell invasion assay was performed using Transwells (8 *μ*m pore size, millipore) with inserts coated with Matrigel (50 mg/mL, BD Biosciences). SW1116 cells (1.0 × 10^5^ cells/well) were seeded in the upper chambers with 0.1 mL matrigel and allowed to invade through matrigel for 16 h. The cells remained on the membranes were fixed with 4% paraformaldehyde and stained with 0.5% crystal violet. The invasive cells and dissolved crystal violet were measured by using microscope and spectrometric absorbance at 570 nm respectively.

### Statistical analysis

All data were analyzed using SPSS 13.0 software (SPSS Inc., Chicago, IL) and expressed as mean ± standard deviation (SD) of repeated experiments in triplicate. The significance of differences was assessed using the Student's *t*‐test. Values of *P* < 0.05 were defined as a statistically significant difference.

## Results

### miR‐145 negatively correlated with PAK4 in several CRC cell lines

**Figure 1:**
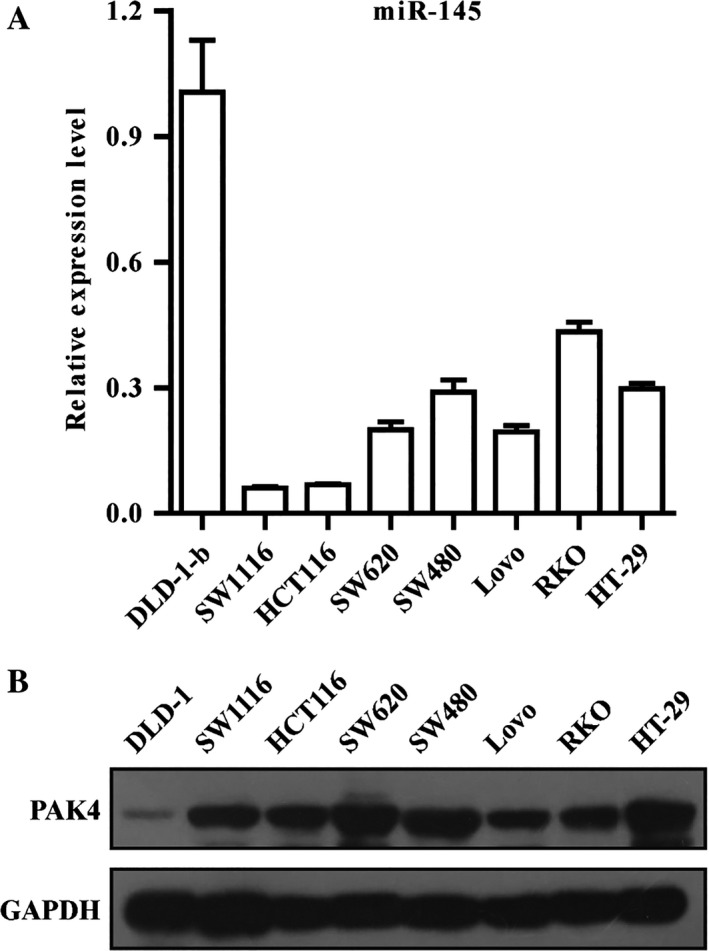
miR‐145 negatively correlated with PAK4 protein levels in CRC cell lines. (A) The mRNA levels of miR‐145 were detected in eight CRC cell lines by qRT‐PCR analysis. (B) The protein expression of PAK4 was detected by western blotting analysis. CRC, colorectal cancer.

To investigate the expression level of miR‐145 in human CRC cells, qRT‐PCR was performed in various cell lines. MiR‐145 was frequently low in multiple CRC cells, HCT116, SW1116, SW620, SW480, Lovo, RKO, and HT29 (Fig. 1A). As a target gene of miR‐145, the protein levels of PAK4 were further confirmed by western blotting (Fig. 1B), which suggest that there was an inverse correlation between miR145 and PAK4 in CRC cells. Based on these results, SW1116 with lower expression of miR‐145 was selected for further study.

### miR‐145 regulate PAK4 expression

**Figure 2:**
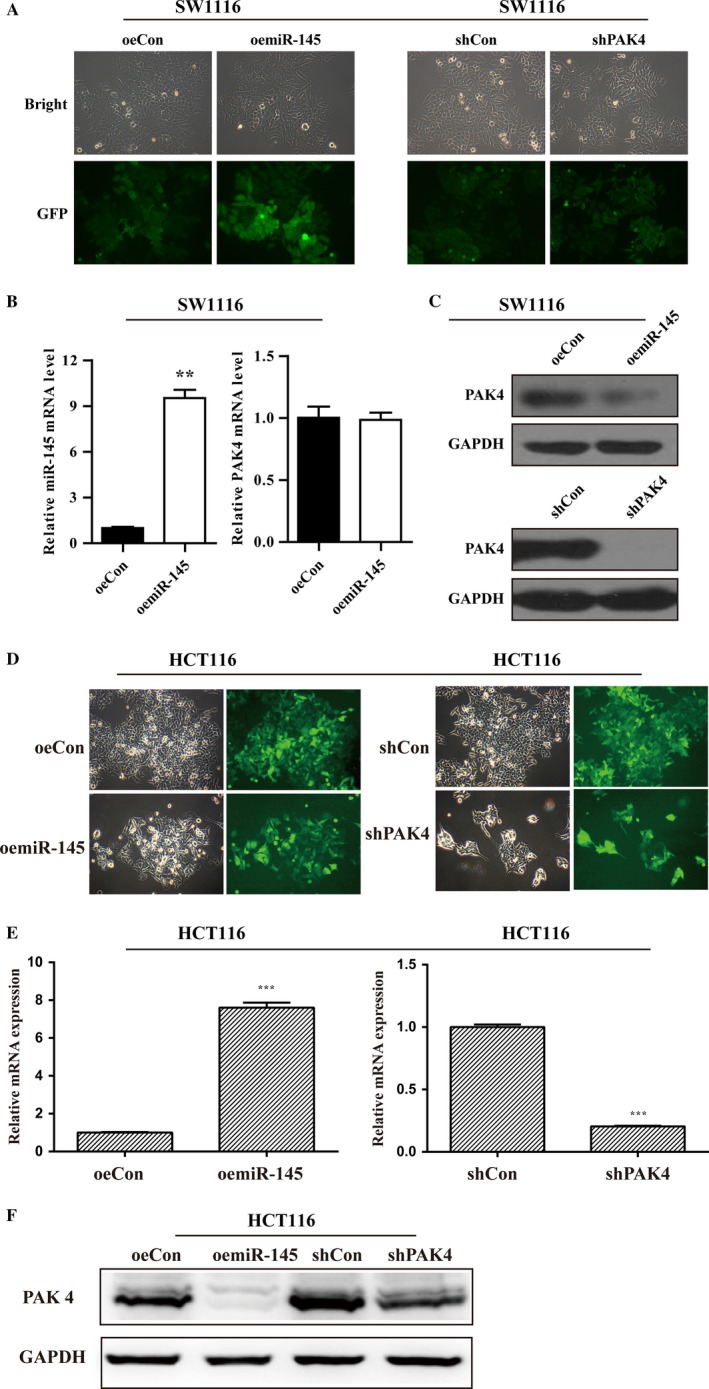
Overexpression of miR‐145 in SW1116 cells inhibited PAK4 protein expression. (A) Microscopic images of SW1116 cells infected with lentivirus. Visible GFP proteins proved that most of cells were successfully infected. (B) qRT‐PCR analysis of miR‐145 overexpression efficiency in SW1116 cells. Overexpression of miR‐145 significantly suppressed the protein levels of PAK4, but not PAK4 mRNA expression. (C) Western blotting analysis of PAK4 knockdown efficiency in SW1116 cells. (D) Microscopic images of HCT116 cells infected with lentivirus. Visible GFP proteins proved that most of cells were successfully infected. (E) qRT‐PCR analysis of miR‐145 overexpression efficiency in HCT116 cells. Overexpression of miR‐145 significantly suppressed the protein levels of PAK4, but not PAK4 mRNA expression. (F) Western blotting analysis of PAK4 knockdown efficiency in HCT116 cells.

Given the roles of miR‐145 and PAK4 in CRC, we carried out experiments for overexpression of miR‐145 and knockdown of PAK4 in SW1116 and HCT116 cells respectively, by infecting the SW1116 and HCT116 cells with lentivirus carrying GFP signals. Majority of SW1116 cells presented GFP‐positive signals (Fig. 2A), suggesting satisfactory infection efficiency. Furthermore, the overexpression and knockdown efficiency were determined using qRT‐PCR analysis and Western blot analysis in SW1116 and HCT116 cells respectively. The mRNA levels of miR‐145 was markedly up‐regulated in SW1116 cells infected with oemiR‐145 (*P* < 0.01) (Fig. 2B), but not that of PAK4 (Fig. 2B). Notably, the protein level of PAK4 was notably suppressed after treated with shPAK4 (Fig. 2C and F). Furthermore, the overexpression of miR‐145 remarkably inhibited PAK4 protein expression compared with that in control(Fig. 2C and F), which suggested that miR‐145 negatively regulated the expression of PAK4 in CRC cells.

### miR‐145 regulate CRC migration and invasion through PAK4

**Figure 3:**
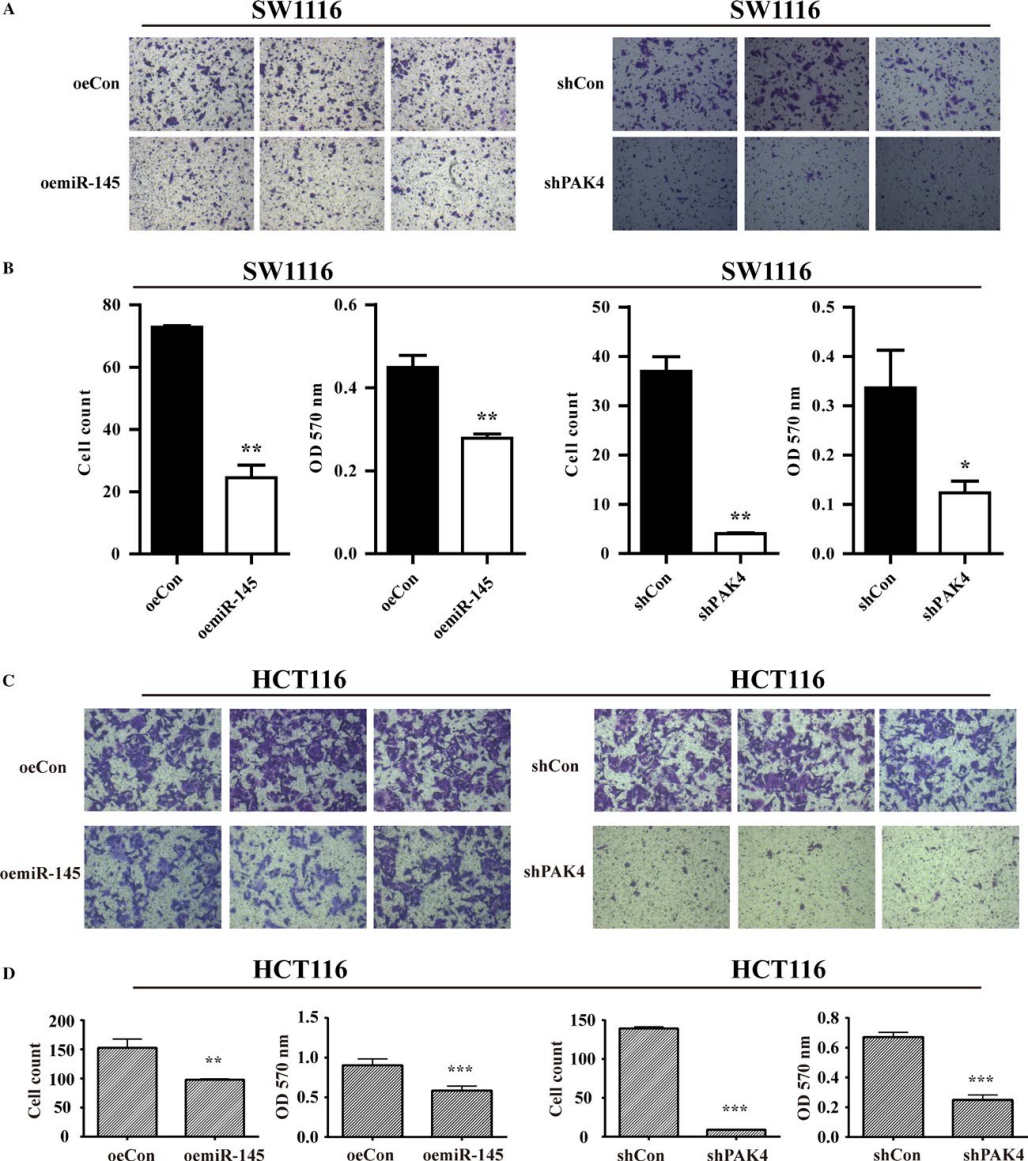
Overexpression of miR‐145 or knockdown of PAK4 suppressed cell migration ability. (A) Representative microscopic photographs of migrated SW1116 cells. Cell counting of migrated SW1116 cells after overexpression of miR‐145 and knockdown of PAK4 from repeated experiments in triplicate. (B) Quantitative analysis of migrated SW1116 cells after overexpression of miR‐145 and knockdown of PAK4 by destaining and reading optical density at 570 nm. (C) Representative microscopic photographs of migrated SW1116 cells. Cell counting of migrated HCT116 cells after overexpression of miR‐145 and knockdown of PAK4 from repeated experiments in triplicate. (D) Quantitative analysis of migrated HCT116 cells after overexpression of miR‐145 and knockdown of PAK4 by destaining and reading optical density at 570 nm. ***P < 0.001,**P < 0.01, *P < 0.05; OD, optical density.

**Figure 4:**
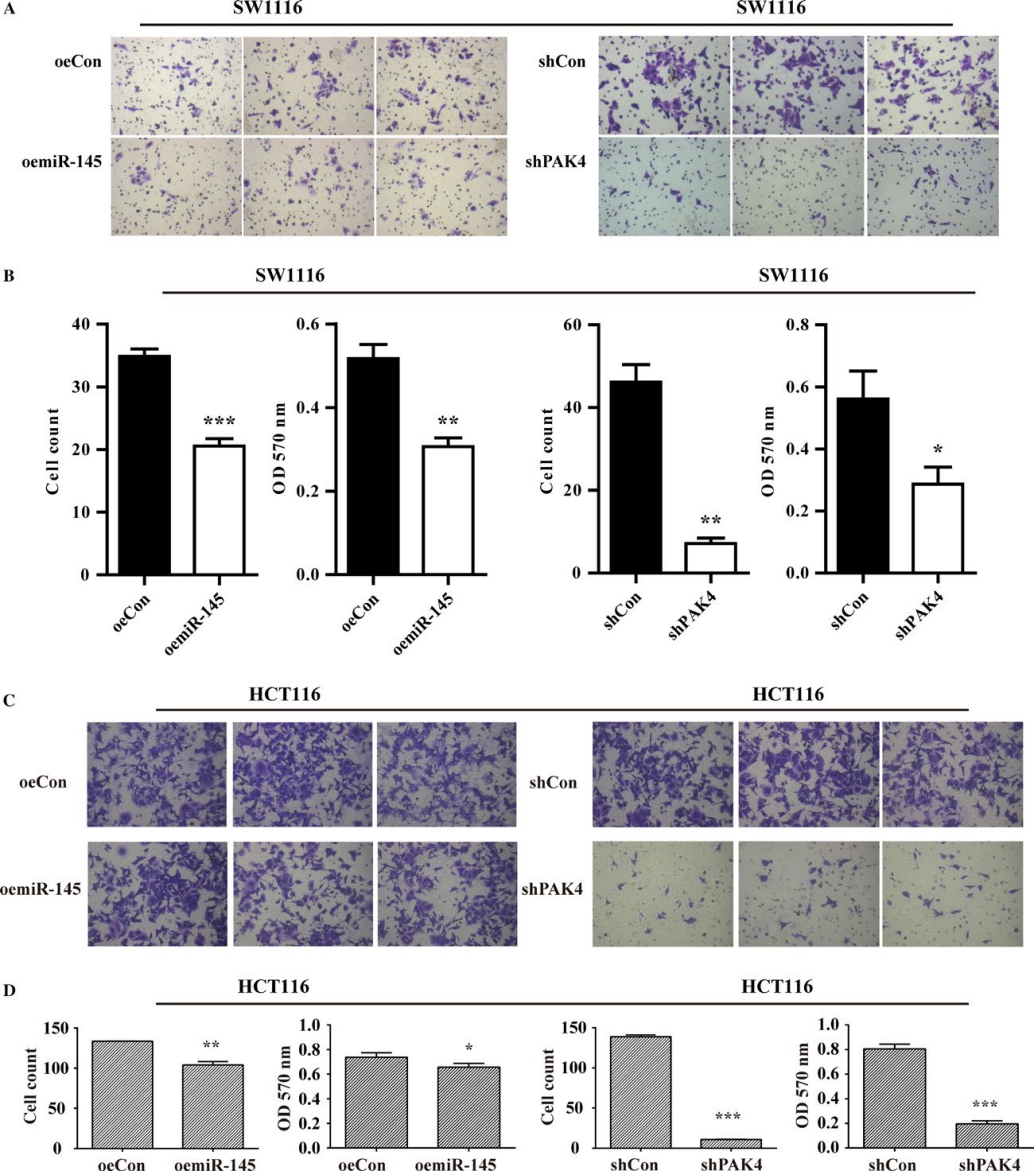
Overexpression of miR‐145 or knockdown of PAK4 suppressed cell invasion ability. (A) Representative three microscopic photographs of migratory SW1116 cells. Cell counting of invasive SW1116 cells after overexpression of miR‐145 and knockdown of PAK4 from repeated experiments in triplicate. (B) Quantitative analysis of invasive SW1116 cells after overexpression of miR‐145 and knockdown of PAK4 by destaining and reading optical density at 570 nm. (C) Representative three microscopic photographs of migratory SW1116 cells. Cell counting of invasive SW1116 cells after overexpression of miR‐145 and knockdown of PAK4 from repeated experiments in triplicate. (D) Quantitative analysis of invasive SW1116 cells after overexpression of miR‐145 and knockdown of PAK4 by destaining and reading optical density at 570 nm. ***P < 0.001,**P < 0.01, *P < 0.05; OD, optical density.

To determine whether miR‐145 contributed to CRC metastasis, repeated transwell assay and matrigel invasion assay experiments in triplicate were carried out to explore the potential biological function of PAK4 and miR‐145 in CRC. As shown in Figure 3A, B, C, and D, overexpression of miR‐145 also caused the reduction of migratory SW1116 and HCT116 cells. Interestingly, PAK4 knockdown leaded to the remarkable reduction of the number and OD values of SW1116 and HCT116 cells penetrating the basement membrane (Fig. 3A, B, C, and D). Furthermore, matrigel invasion analysis showed the numbers and OD values of invasive cells were significantly decreased in oemiR‐145 group compared with that in control (Fig. 4A, B, C, and D). Similar results were also observed in shPAK4 groups (Fig. 4A, B, C, and D). Collectively, miR‐145 and PAK4 may function as a negative and positive regulator in CRC cell migration respectively.

### miR‐145 regulate migration and invasion through LIMK1/cofilin pathway

**Figure 5:**
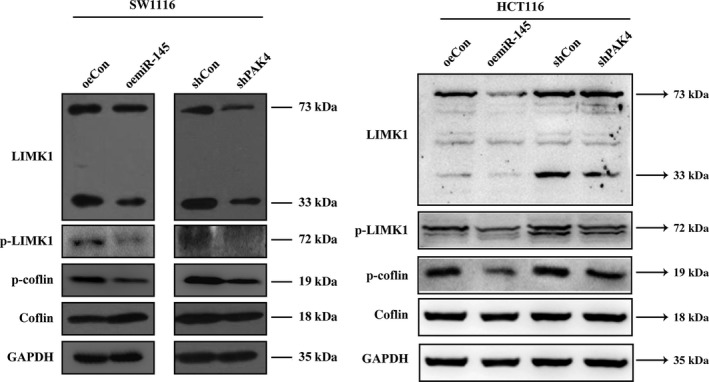
Mechanism by which miR‐145 suppresses migration and invasion in sw1116 cell. Regulation of overexpressed miR‐145 or knockdown of PAK4 on expression of LIMK1, P‐LIMK1, cofilin and P‐cofilin in SW1116 and HCT116 cells, as measured by Western blotting.

It is well‐known that downstream pathways of LIMK1/cofilin were involved in PAK4 regulation of cell migration 17. To further study the mechanism of which PAK4 regulated CRC cells migration and invasion, we detected the protein levels of LIMK1 and cofilin in SW1116 and HCT116 cells. Knockdown of PAK4 or overexpression of miR‐145 significantly decreased the proteins levels of LIMK1 and cofilin (Fig. 5), which indicated that miR‐145 targeted PAK4 to suppress CRC progression might via inhibition of the LIMK1‐conflin signaling pathway.

### High expression of PAK4 is associated with decreased survival

**Figure 6:**
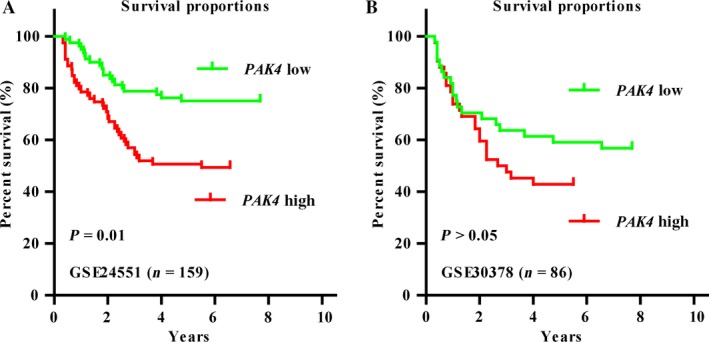
High expression of PAK4 is associated with poor prognosis in cancer patients. (A and B) Kaplan**–**Meyer analysis of overall survival in datasets GSE24551 and GSE30378.

To further explore the correlation between PAK4 and prognosis of CRC patients, we performed the Kaplan**–**Meyer analysis of overall survival in dataset GSE24551 and GSE30378. The results were listed in Figure 6, we concluded from the results that high level of PAK4 resulted in poor prognosis compared with the one with low level (Fig. 6A).However, the difference of PAK4 expression level between the patients who was cured after 2 years was not significant in dataset of GSE30378. This could be due to the small number of patients (Fig. 6B, *n* = 86). There results indicated that PAK4 has a clinical significance in cancer.

## Discussion

Currently, CRC is characterized by rapid metastasis 16 with a poor prognosis. Relative studies have been showed that miRNA participate in most of biological processes, which also included migration and invasion 18. Meantime, miRNA also mediate the occurrence and development of human cancers including CRC 16. Therefore, it is important and necessary to study the specific biological function of miRNA in the process of CRC invasion and metastasis. This can supply a powerful reference for clinic treatment. Recently, it has been demonstrated that miR‐145, as a tumor‐suppressor, participates in CRC tumorigenesis and metastasis. PAK4, one target of miR‐145 16, has been found to be up‐regulated in many cancers and considered as an important oncogene that promotes migration. LIMK1, a downstream effector of PAK4, has been reported to be up‐regulated in lung cancer and associated with high tumor metastasis and lymph node metastasis. However, miR‐145, PAK4 and LIMK1 have not been clearly investigated in the CRC invasion and metastasis.

Here, we demonstrated that miR‐145 expression was reversely correlated with PAK4 in CRC cells, in concordance with previous studies 16, 18. To discuss function of miR‐145 and PAK4 in CRC cell migration, SW1116 cells with lower miR‐145 and higher PAK4 expression, was chosen to perform functional analysis. We constructed SW1116 stable cell lines which overexpressed miR‐145 and PAK4‐silencing, respectively, and found both of these constructed cells presented impaired migration and invasion ability. Our results further revealed that overexpression of miR‐145 or knockdown of PAK4 significantly decreased the expression of LIMK1, as well as the ability of LIMK1 to phosphorylate cofilin. In addition, miR‐145 has been demonstrated targets a putative binding site in the 3’UTR of PAK4 by using luciferase reporter assay in CRC 16. Thus, miR‐145 might regulate cell migration and invasion by targeting PAK4 in a direct way to affect LIMK1 and cofilin in CRC metastasis.

PAK4 is a subfamily of serine/threonine kinases which participate in the regulation of cell motility and cytoskeletal dynamics. It has been reported cytoskeletal organization has an irreplaceable function in most cellular motility 19. However, signaling enzymes which involve in the regulation of cytoskeleton usually result in harmful effect if they are regulated in an improper way 20, 21, 22. Previous studies have demonstrated that PAK4 and LIMK1 could lead to cytoskeletal changes in C2C12 cells together. Moreover, activated PAK4 stimulated LIMK1 to phosphorylate cofilin in regulating cytoskeletal changes using immune complex kinase assays 23. Based on these evidences, the mechanisms of PAK4, involved in LIMK1‐cofilin signaling pathway, might be linked to CRC cell migration.

PAK4 regulates cell migration mainly dependent on the downstream pathways of LIMK1/cofilin in prostate cancer and gastric cancer. And the PAK4‐LIMK1‐cofilin signaling pathway promotes cell migration in these two cancers 24. Overexpression of LIMK1 has been reported to be associated with higher tumor metastasis stage and lymph node metastasis 25. Our finding is consistent with previous report that knockdown of LIMK1 can suppress the migration and invasion of lung cancer cells 25. As substrate of LIMK1, cofilin plays an important role in depolymerizing filamentous actin (F‐actin) 25, 26 and defining the direction of cell motility 27. Collectively, these results indicated that knockdown of PAK4 by miR‐145 could downregulate the LIMK1‐cofilin pathway and inhibit F‐actin mediated cell migration of CRC cells. This is the initial study to exhibit the molecular mechanism that PAK4 is negatively regulated by miR‐145 in inhibiting cell migration of CRC.

The Kaplan**–**Meyer analysis of overall survival indicated that high level of PAK4 was significantly associated with poor prognosis in CRC patients. However, whether low expression of miR‐145 could predict poor prognosis in CRC patients has not been obtained. The relative study demonstrated that lower expression of miR‐145 resulted in tumors with maximal tumor diameter miR‐145 (*P* = 0.003) in colorectal cancer. In addition, some relative studies found lower expression of miR‐145 predict poor prognosis in different cancer patients. For example, Shen et al. 28 found that low miR‐145 expression level is associated with poor prognosis in non‐smal cell lung cancer. Also, downregulation of miR‐145‐5p correlated with poor prognosis in gastric cancer 29. The same conclusion was also obtained in oral squamous cell carcinomas 30, cervical cancer 31, osteosarcoma 32, and prostate cancer 33.These results convinced us that lower expression of miR‐145 may predict poor prognosis. And a further study is needed to demonstrate this conclusion.

In summary, the present study demonstrates that miR‐145 plays an important role in inhibiting cell migration by directly targeting PAK4, and identifies miR‐145‐PAK4‐LIMK1‐cofilin as a novel regulatory pathway that contributed to CRC metastasis. High expression of PAK4, a direct target of miR‐145, was significantly associated with poor prognosis in CRC patients, indicating that miR‐145 might be a new predictor for CRC prognosis. Our study indicates that miR‐145‐PAK4‐LIMK1‐cofilin signaling pathway might be related to the progression of CRC and provides an effective molecular target for CRC diagnosis and therapy.

## Conflict of Interest

None declared.
