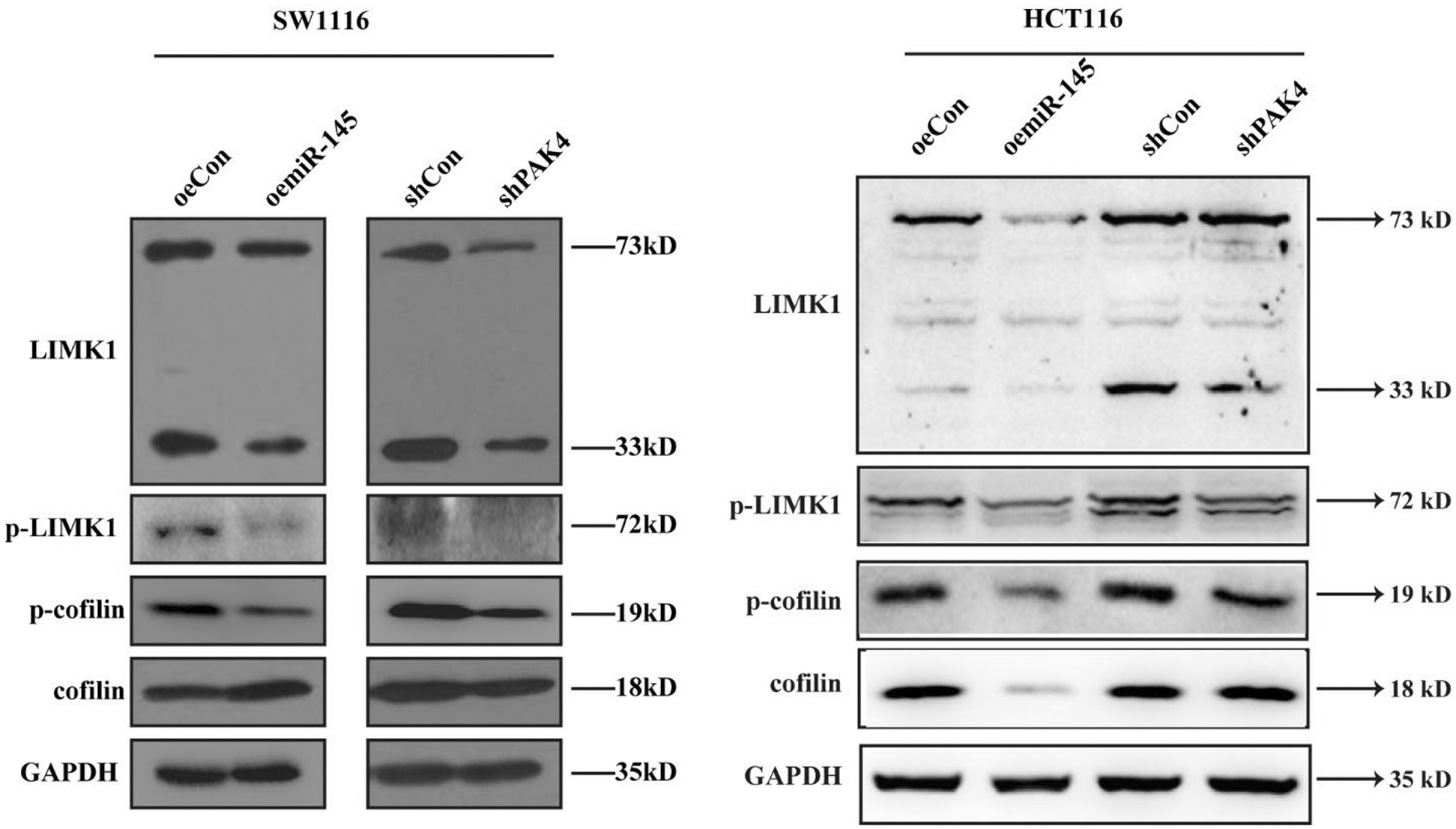# Correction to “MiR‐145 inhibits human colorectal cancer cell migration and invasion via PAK4‐dependent pathway”

**DOI:** 10.1002/cam4.7386

**Published:** 2024-06-25

Nengquan Sheng, Gewen Tan, Weiqiang You, Hongqi Chen, Jianfeng Gong, Di Chen, Huizhen Zhang, Zhigang Wang. MiR‐145 inhibits human colorectal cancer cell migration and invasion via PAK4‐dependent pathway. *Cancer Medicine* 2017; 6(6): 1331–1340. https://doi.org/10.1002/cam4.1029.

An investigation based on concerns raised by a third party revealed the duplication of the cofilin and GAPDH lane in Figure 5, HCT116 panel. The authors admitted to the image compilation error and were able to provide the original images. The authors confirm that all the experimental results and corresponding conclusions mentioned in the paper remain unaffected and sincerely apologize for this mistake. The corrected Figure 5 is shown as follows.

**Corrected Figure 5:**